# Salvage Surgery Including Rectal Wall Vas Deferens Pexy for Recurrent Bilateral Perineal Hernia with Bladder Retroflexion in a Dog: A Case Report

**DOI:** 10.3390/vetsci13090969

**Published:** 2026-09-16

**Authors:** Junyeong Park, Jaewon Do, Changmin Park, Seohyeon Yoon, Ki-Dong Eom, Hwi-Yool Kim

**Affiliations:** 1 Department of Veterinary Surgery, College of Veterinary Medicine, Konkuk University, Neungdongro 120, Seoul 05029, Republic of Korea; jy0225@konkuk.ac.kr (J.P.); wn16859@naver.com (J.D.); cmpark9575@konkuk.ac.kr (C.P.); 2 Department of Veterinary Medical Imaging, College of Veterinary Medicine, Konkuk University, Seoul 05029, Republic of Korea; ysh0701@konkuk.ac.kr (S.Y.); eomkd@konkuk.ac.kr (K.-D.E.)

**Keywords:** perineal hernia, bladder retroflexion, vas deferens pexy, rectal wall fixation, adhesiolysis, pelvic diaphragm reconstruction, salvage surgery, dog

## Abstract

Bladder retroflexion associated with perineal hernia may cause urinary dysfunction, and surgical correction can be challenging after repeated procedures because of extensive adhesions and altered anatomy. An 11-year-old, 7.5 kg, castrated male Pomeranian was referred for recurrent bilateral perineal hernia, bladder retroflexion, and persistent urinary dysfunction after multiple perineal and abdominal surgeries. A previous attempt at abdominal bladder reduction and cystopexy had failed because adhesions prevented adequate mobilization of the bladder. During salvage surgery, bilateral perineal adhesiolysis was performed first to release the displaced urinary tract structures, followed by an abdominal approach to restore the bladder to its normal intra-abdominal position. Modified bilateral vas deferens pexy was then performed by fixing the residual vasa deferentia to the rectal wall, and each side of the pelvic diaphragm was reconstructed according to the available tissues and defect configuration. Retrograde urethrocystography performed 8 days postoperatively demonstrated short-term anatomical correction, with restoration of the normal positions of the bladder, bladder neck, and urethra. Urinary function gradually improved but had not completely normalized by 150 days. Without further objective imaging, long-term mechanical stability and prevention of recurrence could not be established. This case describes a multimodal salvage strategy for recurrent bilateral perineal hernia with markedly altered anatomy after repeated surgery.

## Simple Summary

Perineal hernia occurs when tissues that normally support organs in the pelvic region become weakened, allowing organs such as the urinary bladder to move into the area beside the anus. Surgical treatment can become especially difficult after repeated operations because scar tissue and adhesions can change the normal anatomy. This report describes an 11-year-old Pomeranian that had undergone several previous surgeries but continued to have recurrent hernias, abnormal bladder displacement, and urinary problems. During the final corrective surgery, adhesions were first released through the perineal region to free the bladder and nearby urinary structures. The bladder was then returned to its normal abdominal position, a modified fixation technique was used to reduce the risk of it moving backward again, and the weakened tissues on both sides of the pelvis were repaired according to the tissues available. Imaging performed 8 days after surgery demonstrated short-term anatomical correction, with the bladder and bladder neck in their normal positions and the urethra following a normal course. Urinary function improved gradually but had not completely returned to normal by 150 days after surgery. This case illustrates how surgical techniques can be adapted and combined when repeated operations have substantially altered the normal anatomy.

## 1. Introduction

Perineal hernia results from weakening or disruption of the pelvic diaphragm, allowing pelvic or abdominal structures to displace into the perineal region, and occurs predominantly in middle-aged to older male dogs [1,2]. Hernial contents may include adipose tissue, rectum, prostate, and urinary bladder [1,2]. In particular, retroflexion or caudal displacement of the urinary bladder may be associated with urinary complications such as dysuria or urethral obstruction [2,3,4,5]. Therefore, in perineal hernias involving the urinary bladder, treatment goals may include not only reconstruction of the pelvic diaphragm but also reduction and stabilization of displaced urinary tract structures [6,7].

Surgical treatment of perineal hernia is primarily based on reduction in the hernial contents and reconstruction of the pelvic diaphragm. Several reconstructive techniques have been described, with internal obturator muscle transposition being one of the most commonly reported methods [8,9,10,11]. However, in complex cases involving bilateral herniation or displacement of pelvic organs such as the urinary bladder and prostate, correction of the anatomical abnormalities may be difficult using a perineal approach alone [6,7]. In such cases, an abdominal approach may be combined with perineal repair to reduce displaced organs, and organopexy may be added when necessary to limit recurrent displacement [6,7].

Previous abdominal surgery can result in postoperative adhesion formation, which may complicate subsequent surgical procedures [12]. Previous perineal surgery may likewise be associated with scarring and alteration of local urinary tract anatomy [13]. In complex revision cases involving both regions, these changes may complicate the identification of, dissection of, and reduction in displaced urinary tract structures. In recurrent cases with previous surgical intervention, the condition of the available local tissues and the effects of prior procedures may also limit the direct application of conventional pelvic diaphragm reconstructive techniques [11,14,15]. Therefore, complex revision cases may require a salvage strategy in which multiple surgical techniques are selectively combined or modified according to the intraoperative anatomy, available tissues, and technical constraints [6,14,15].

To address these anatomical and technical challenges, a multimodal salvage surgical strategy was adopted in the present case. Perineal adhesiolysis was performed before abdominal bladder reduction, and modified bilateral vas deferens pexy to the rectal wall was used to prevent recurrent bladder retroflexion. In addition, each side of the pelvic diaphragm was reconstructed according to the available tissues and configuration of the defects. The purpose of this case report was to describe the surgical management of recurrent bilateral perineal hernia with bladder retroflexion in the setting of markedly altered anatomy and to report the short-term anatomical outcome and postoperative functional course.

## 2. Case Description

### 2.1. Patient Information and History

An 11-year-old, 7.5 kg, castrated male Pomeranian was referred for recurrent bilateral perineal swelling, dehiscence of a left perianal surgical wound, and persistent urinary retention requiring an indwelling urinary catheter. The dog had undergone four operative episodes before referral, as described chronologically below. Approximately 3–4 years before referral, the dog had undergone bilateral perineal hernia repair and castration at a local veterinary hospital. Detailed records regarding the techniques and suture materials used for the previous hernia repairs were unavailable.

Twenty-five days before referral, the dog was presented to a local veterinary hospital for a mass located immediately ventral to the anus. Physical examination revealed perineal swelling, and diagnostic imaging identified recurrence of a left perineal hernia containing the urinary bladder. Excision of the perianal mass and repair of the left perineal hernia using polypropylene mesh were performed. Histopathological examination of the excised mass revealed a hepatoid gland carcinoma with incomplete surgical margins. Tumor staging was not performed, and no further oncological treatment was undertaken. Approximately 2 h after the left perineal hernia repair, a recurrent right perineal hernia was identified. Twenty-three days before referral, the right perineal hernia was repaired using polypropylene mesh. Following surgery, the dog was unable to urinate spontaneously, and an indwelling urinary catheter was placed for management of urinary retention. Subsequent diagnostic imaging demonstrated pelvic displacement of the urinary bladder.

Eight days before referral, abdominal reduction of the bladder and cystopexy were first attempted. However, extensive adhesions between the bladder and surrounding tissues prevented adequate dissection and manipulation, and bladder reduction and cystopexy could not be completed. The previous left perineal surgical site was subsequently reopened, the previously placed mesh was removed, and adhesiolysis was performed; however, the bladder could not be returned to its normal anatomical position. The dog was subsequently referred with the indwelling urinary catheter still in place.

### 2.2. Clinical Findings

At referral, the dog weighed 7.5 kg and had a body condition score of 8/9. The dog was quiet, alert, and responsive. Rectal temperature was 39.7 °C, heart rate was 105 beats/min, and respiration consisted of panting. Non-invasive systolic blood pressure was 156 mmHg. The mucous membranes were pink, with a capillary refill time of <1 s. No abnormalities were detected on cardiac or pulmonary auscultation.

Physical examination revealed bilateral non-reducible perianal masses and fecal incontinence. The right perineal mass measured approximately 4 × 7 × 1.5 cm and was firm and non-mobile. No obvious local heat, erythema, or additional swelling was detected around the mass. The left perineal mass measured approximately 4 × 7 × 2 cm and was also firm and non-mobile. Partial dehiscence of the previous surgical site with persistent hemorrhagic discharge was observed, together with mild local heat, erythema, and swelling. Fine-needle aspiration of the left perineal mass revealed low-cellularity pyogranulomatous inflammation and spindle-shaped fibroblasts, consistent with chronic inflammation and tissue repair. Fine-needle aspiration of the right perineal mass revealed adipocytes and fragments of adipose tissue.

Hematological examination revealed leukocytosis (27.13 × 10^3^/μL) and a mildly decreased packed cell volume of 35.8%. Blood urea nitrogen and serum creatinine concentrations were 11 mg/dL and 1.3 mg/dL, respectively, and were within their reference intervals. The symmetric dimethylarginine concentration was mildly increased at 17 μg/dL. The C-reactive protein concentration was markedly increased at 8.9 mg/dL.

### 2.3. Diagnostic Imaging

**Figure 1:**
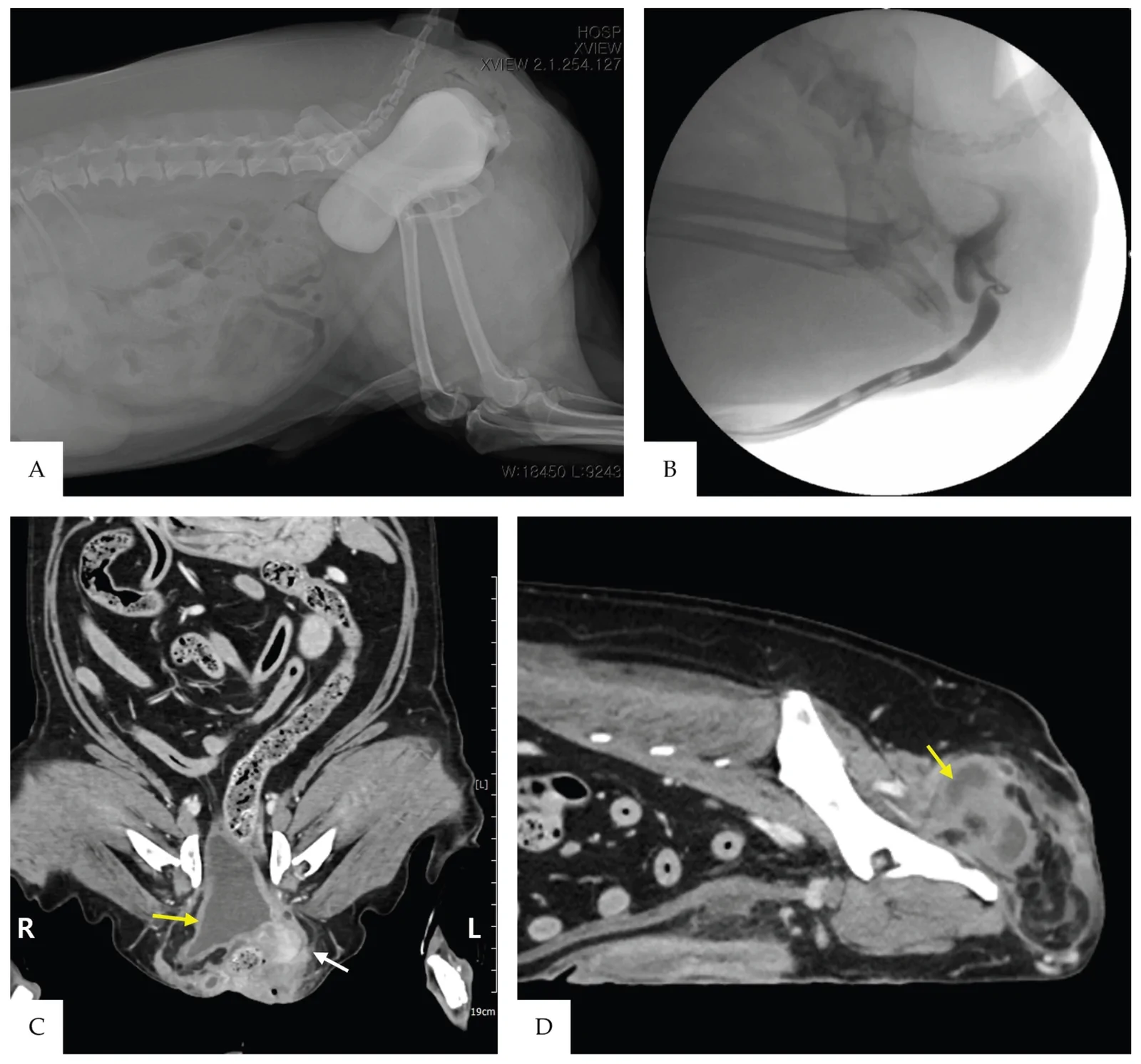
Preoperative imaging findings of the urinary bladder and urethra. (**A**) Lateral cystographic image showing marked caudal displacement of the urinary bladder into the pelvic/perineal region. (**B**) Fluoroscopic urethrocystographic image demonstrating caudal deviation of the urethra with focal luminal narrowing near the prostate (yellow arrowhead). (**C**,**D**) Computed tomography images showing displacement of the urinary bladder into the perineal region (yellow arrows) and leftward deviation of the internal urethral orifice (white arrow).

Cystography performed at the referring hospital 14 days before referral demonstrated caudal displacement of the urinary bladder from its normal abdominal position into the pelvic cavity (Figure 1A). On precontrast abdominal radiographs obtained at referral, the urinary bladder silhouette was not visible in its normal anatomical location. Retrograde urethrocystography demonstrated marked displacement of the urinary bladder toward the perineal region. The urethra was also deviated caudally from its normal course, and focal narrowing of the urethral lumen was observed adjacent to the prostate (Figure 1B).

Abdominal ultrasonography revealed mild bilateral renal pelvic dilation, measuring 4.7 mm on the right and 5.0 mm on the left. Computed tomography demonstrated retroflexion of the urinary bladder through the pelvic cavity into the perineal region. The bladder neck and ureters were displaced into the left perineal hernia, and the internal urethral orifice was deviated to the left (Figure 1C,D). The right perineal mass was predominantly of fat attenuation, with surrounding fat stranding. The urinary bladder wall was mildly thickened, with a maximum thickness of approximately 4.0 mm.

Based on the clinical and imaging findings, recurrent bilateral perineal hernia with bladder retroflexion, displacement of the bladder neck and ureters into the left perineal hernia, focal urethral luminal narrowing adjacent to the prostate, and decreased anal sphincter function were diagnosed. Surgical site infection of the left perineal wound was suspected.

### 2.4. Preoperative Management and Surgical Planning

Definitive corrective surgery was delayed for 2 weeks after referral to allow the dehiscence and local inflammation of the left perineal surgical site to resolve. During this period, the indwelling urinary catheter was maintained. A sample from the left perineal surgical site was submitted for bacterial culture and antimicrobial susceptibility testing; however, no bacterial growth was detected, precluding susceptibility testing. Because surgical site infection remained clinically suspected, empirical antimicrobial therapy with amoxicillin–clavulanate (13.75 mg/kg PO every 12 h) was administered for 14 days. At re-evaluation 14 days after referral, wound dehiscence, discharge, and local inflammatory changes had clinically resolved. The C-reactive protein concentration had decreased from 8.9 mg/dL at referral to 0.3 mg/dL.

The main surgical objectives were to separate the urinary bladder, bladder neck, ureters, and urethra from the extensive surrounding adhesions; restore the urinary bladder to its normal intra-abdominal position; prevent recurrent bladder retroflexion; and reconstruct the bilateral pelvic diaphragms. Pubic osteotomy was considered as a contingency if sufficient exposure of the bladder and urethra could not be obtained through the perineal and abdominal approaches.

### 2.5. Surgical Treatment

Surgery was performed 15 days after referral. Amoxicillin–clavulanate (13.75 mg/kg) and metronidazole (15 mg/kg) were administered intravenously before surgery. Premedication consisted of midazolam (0.2 mg/kg IV), fentanyl (2 μg/kg IV), and maropitant (1 mg/kg SC). General anesthesia was induced with propofol (6 mg/kg IV) and maintained with isoflurane. A continuous intravenous infusion of fentanyl was administered for intraoperative analgesia.

#### 2.5.1. Perineal Approach and Adhesiolysis

**Figure 2:**
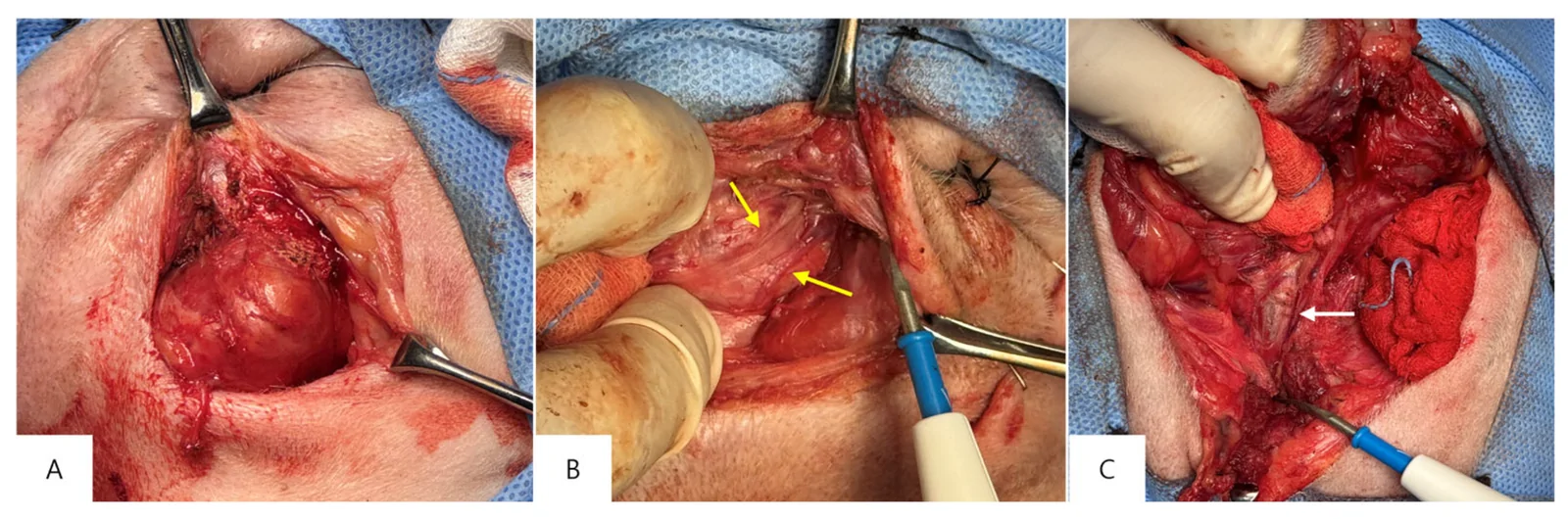
Intraoperative findings during perineal adhesiolysis. (**A**) Right perineal hernia site showing the perineal mass and previously implanted polypropylene mesh. (**B**) Left perineal hernia site showing the ureters (yellow arrow) during adhesiolysis. (**C**) Urethra identified during further perineal adhesiolysis (white arrow).

The dog was positioned in sternal recumbency. A right perianal incision was made to expose the right perineal mass and previously implanted polypropylene mesh (Figure 2A). The mesh adherent to the mass was separated, and the mass was dissected from the surrounding tissues. The right incision was then temporarily closed with skin staples.

The previous left perianal surgical incision was reopened, and the ureters and urinary bladder were carefully separated from the surrounding adhesions (Figure 2B). The balloon of the urethral catheter was palpated and used as an anatomical landmark for the bladder neck. To improve exposure of the displaced urinary tract structures, an additional incision was made ventral to the anus to connect the bilateral perianal incisions. Further adhesiolysis around the urinary bladder and urethra was performed through the connected incision (Figure 2C). After completion of the perineal adhesiolysis, the connected incision was temporarily closed with skin staples.

#### 2.5.2. Bladder Reduction and Bilateral Vas Deferens Pexy

**Figure 3:**
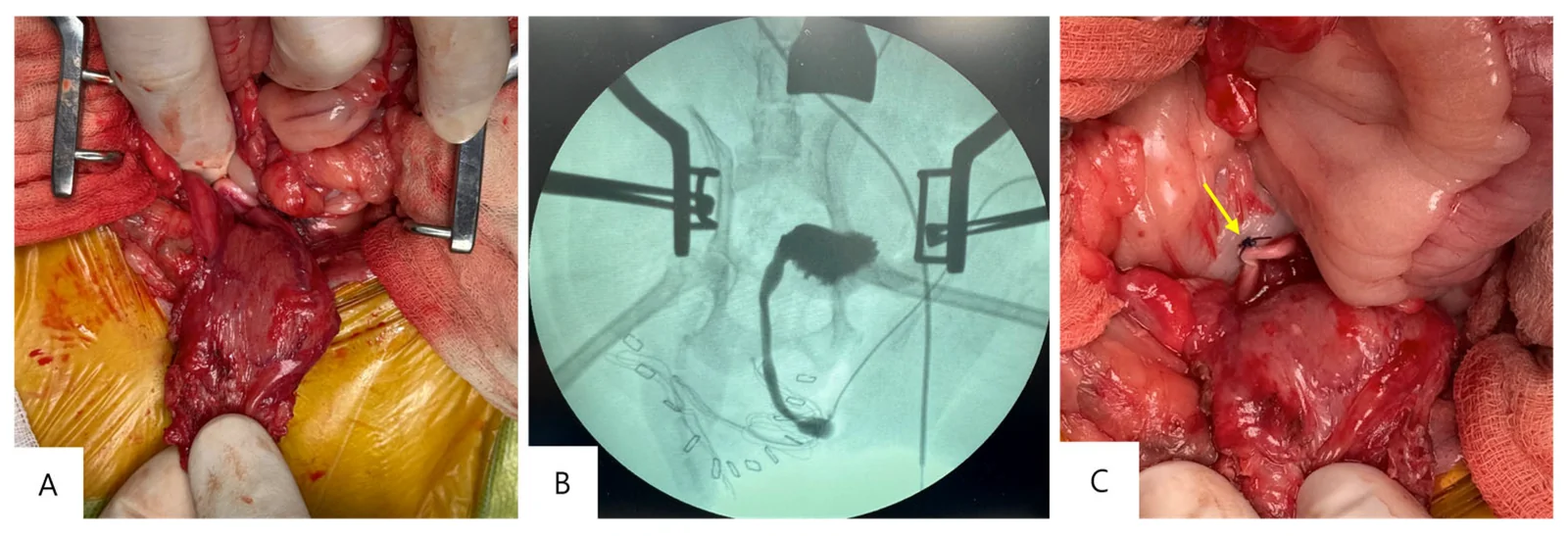
Intraoperative images of abdominal bladder reduction and vas deferens pexy. (**A**) Urinary bladder sufficiently mobilized to permit reduction. (**B**) Intraoperative urethrocystographic image obtained after bladder reduction to assess urethral patency. (**C**) Fixation of the remnant vas deferens to the rectal wall (yellow arrow).

The dog was repositioned in dorsal recumbency, and a caudal ventral midline celiotomy was performed. Following the preceding perineal adhesiolysis, the urinary bladder was sufficiently mobilized to permit reduction (Figure 3A). The right perineal mass adherent to the urinary bladder was then dissected and excised, after which the bladder was restored to its normal intra-abdominal position.

After bladder reduction, intraoperative retrograde urethrocystography was performed to assess urethral patency (Figure 3B). To prevent recurrent bladder retroflexion, bilateral vas deferens pexy was performed using the residual vasa deferentia. Each residual vas deferens was fixed to the ventral aspect of the cranial rectum using 3-0 polydioxanone (Figure 3C). On each side, a single mattress suture incorporated two partial-thickness rectal-wall bites and two intervening full-thickness passes through the vas deferens. The rectal-wall bites were oriented transversely to the longitudinal axis of the rectum. To minimize the risk of rectal luminal penetration, the needle was advanced tangentially through the rectal wall, incorporating the muscular layer without entering the lumen. The abdominal incision was routinely closed after completion of the vas deferens pexy.

#### 2.5.3. Bilateral Perineal Hernia Repair

After completion of the abdominal procedure, the dog was repositioned in sternal recumbency. The contents of the left perineal hernia were reduced, and internal obturator muscle transposition was performed to reconstruct the left pelvic diaphragm. On the right side, residual internal obturator muscle tissue was identified centrally and preserved as part of the reconstruction. The pelvic diaphragm defects on either side of the residual muscle were closed by apposition of the surrounding residual perineal tissues, including the external anal sphincter and coccygeus muscle, using 3-0 polydioxanone in a simple interrupted pattern. The sacrotuberous ligament was incorporated into selected lateral sutures for additional reinforcement. To minimize the risk of sciatic nerve entrapment or injury, sutures engaging the sacrotuberous ligament were passed through the substance of the ligament rather than around it. Disrupted portions of the external anal sphincter and retractor penis muscle were reapproximated and sutured in their anatomical positions. The remaining soft tissues and skin were closed routinely in layers. Histopathological examination of the excised right perineal mass revealed adipose tissue with traumatic changes.

### 2.6. Postoperative Course and Follow-Up

Postoperatively, amoxicillin–clavulanate (13.75 mg/kg IV every 12 h) was continued through postoperative day 7, and metronidazole (15 mg/kg IV every 12 h) was administered through postoperative day 3. Postoperative analgesia consisted of a fentanyl continuous rate infusion, initiated at 3 μg/kg/h and subsequently reduced to 2 μg/kg/h, which was continued through postoperative day 3. After discontinuation of fentanyl, meloxicam (0.2 mg/kg SC) was administered, followed by 0.1 mg/kg SC every 24 h through postoperative day 7. Pain was assessed using the Glasgow Composite Measure Pain Scale–Short Form (CMPS-SF), with a score of ≥6/24 used as the criterion for rescue analgesia.

**Figure 4:**
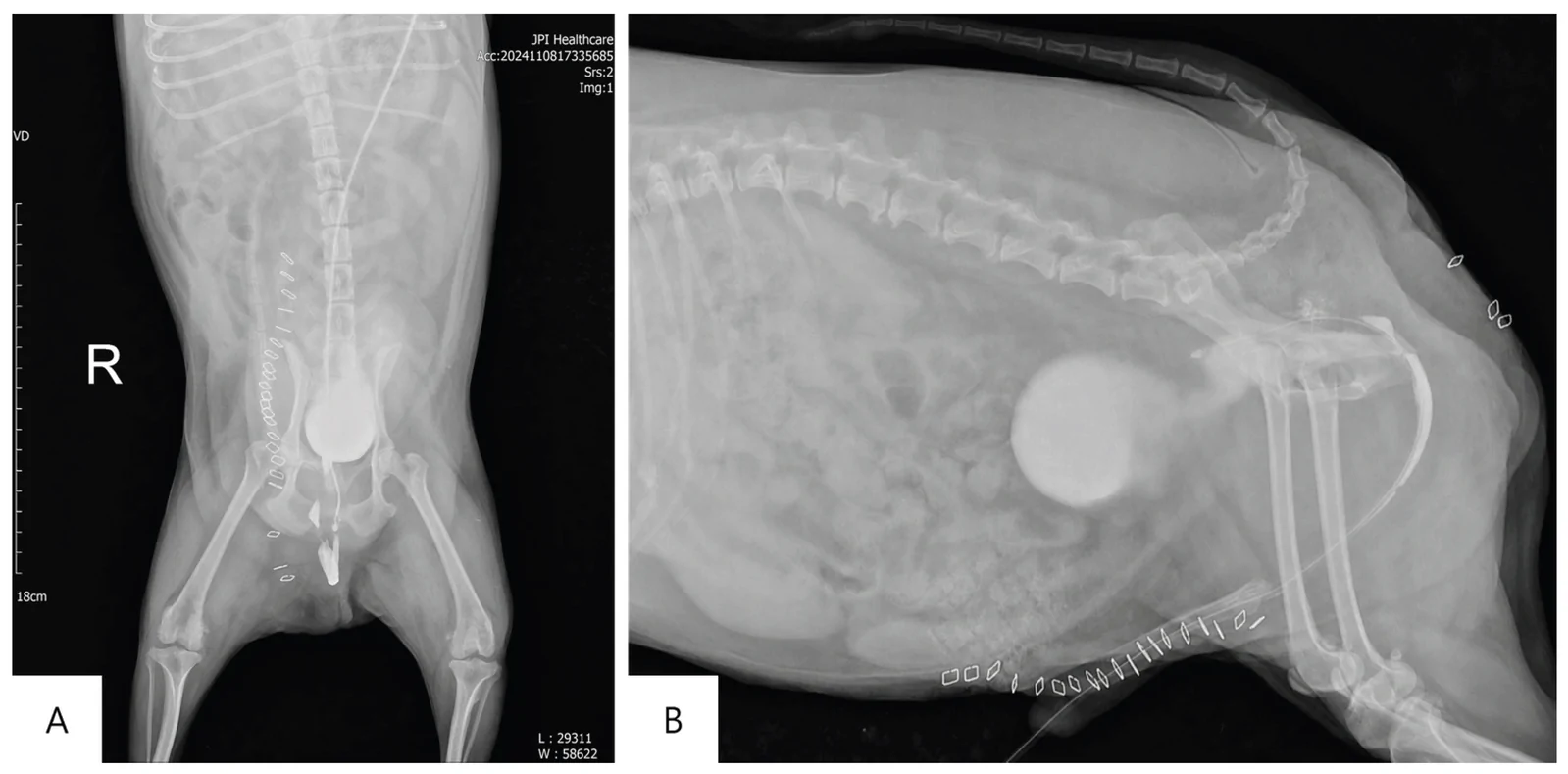
Postoperative urethrocystography on postoperative day 8. (**A**) Ventrodorsal and (**B**) left lateral views showing normal positioning of the urinary bladder, bladder neck, and urethra.

The indwelling urinary catheter was maintained postoperatively and removed on postoperative day 3. Following catheter removal, urinary incontinence was observed. The dog attempted spontaneous urination, but the urinary stream was weak and voiding remained difficult. Fecal incontinence also persisted. Retrograde urethrocystography performed on postoperative day 8 showed that the urinary bladder was maintained in its normal intra-abdominal position, with the bladder neck in a normal position and the urethra following a normal anatomic course. No recurrent displacement of the urinary bladder into the pelvic cavity or perineal region was identified (Figure 4).

The dog was discharged on postoperative day 9 for continued treatment and monitoring at the referring veterinary hospital. Subsequent follow-up was conducted by telephone with the owner and the referring veterinarian, and dysuria and urinary incontinence were assessed qualitatively based on their observations. At 30 days postoperatively, the dog was able to urinate spontaneously; however, dysuria and urinary incontinence persisted. Fecal incontinence was also still present. At 150 days postoperatively, urinary function had gradually improved but had not completely returned to normal. In contrast, fecal incontinence had markedly improved compared with the immediate postoperative period.

## 3. Discussion

This case describes the use of a combined perineal and abdominal approach, with perineal adhesiolysis performed before abdominal bladder reduction, together with modified vas deferens pexy in a dog with recurrent bilateral perineal hernia, extensive adhesions, and bladder retroflexion following multiple previous perineal and abdominal surgeries. Because detailed records of the previous procedures were unavailable, the exact cause of recurrence and the contribution of each previous procedure to the subsequent anatomical changes could not be determined. Recurrent perineal hernia after previous repair may be associated with compromised pelvic diaphragm tissues, which can complicate revision surgery [11,14,15]. In the present case, extensive adhesions and marked alteration of normal anatomical relationships were encountered during revision surgery. Urinary retention had occurred after the previous right perineal hernia repair, and caudal displacement of the bladder neck and urethra was subsequently identified. Caudal bladder displacement can result in urethral kinking and dynamic obstruction [4,5], and similar anatomical abnormalities may have contributed to the urinary dysfunction observed in this dog. Because dehiscence and local inflammation were present at the left surgical site at referral, definitive corrective surgery was performed after these abnormalities had clinically resolved.

In bilateral or complicated perineal hernias, a staged procedure consisting of initial laparotomy to reduce displaced organs and perform organopexy, including colopexy, vas deferens pexy, or cystopexy when indicated, followed by perineal reconstruction has been described [6,7]. In the present case, however, abdominal bladder reduction and cystopexy had already been attempted before referral, but extensive adhesions between the bladder and surrounding tissues prevented adequate mobilization and reduction. Bilateral perineal adhesiolysis was therefore performed first to release the displaced urinary tract structures before an abdominal approach was used to restore the urinary bladder to its normal intra-abdominal position. Because exposure through the bilateral perianal incisions alone remained limited, the incisions were connected ventral to the anus to improve access for adhesiolysis. A ventral semicircular perineal incision has been reported to improve surgical exposure in dogs with bilateral perineal hernia and severe organ prolapse [16]. The sequence used in the present case should not be considered an alternative standard approach; rather, perineal adhesiolysis before abdominal reduction was selected because of the distribution of the adhesions and the inability to reduce the bladder during the previous abdominal procedure.

After bladder reduction, bilateral vas deferens pexy was performed instead of direct cystopexy. Vas deferens pexy has been described for correction of caudal displacement of the urinary bladder and prostate in dogs with perineal hernia, with the conventional technique involving fixation of both vasa deferentia to the lateral abdominal wall [17]. The procedure has subsequently been used as part of abdominal surgical management for complicated perineal hernia [6,7] and has also been reported in a previously castrated dog when sufficient residual vas deferens length was available [14]. In the present case, bladder retroflexion was accompanied by caudal displacement of the bladder neck and an abnormal urethral course. Therefore, indirect cranial stabilization using the vasa deferentia was considered likely to help limit recurrent caudal displacement of the bladder neck and maintain a more normal urethral course. In addition, because the urinary bladder had already undergone repeated manipulation and extensive adhesiolysis, an indirect stabilization technique that avoided an additional fixation procedure involving the bladder wall was selected. This choice should not be interpreted as indicating superiority of vas deferens pexy over cystopexy. Cystopexy has also been reported to successfully correct caudal displacement of the urinary bladder and associated urethral kinking, with postoperative contrast cystourethrography confirming cranial repositioning of the bladder and straightening of the urethra [5].

Unlike the conventional technique, the residual vasa deferentia in the present case were fixed to the rectal wall. Intraoperatively, the available length of each residual vas deferens was considered insufficient for tension-free fixation to the lateral abdominal wall without further mobilization. Application of the conventional technique would therefore have required additional dissection and cranial and lateral traction of the vasa deferentia. In contrast, the rectal wall was anatomically closer to the bladder neck and prostate and had already been exposed during pelvic adhesiolysis, allowing fixation over a shorter distance. The rectal wall was therefore selected as the fixation site to avoid additional mobilization of the vasa deferentia and dissection toward the abdominal wall and to minimize further extension of an already extensive surgical procedure. This rectal wall vas deferens pexy should be interpreted as a patient-specific modification performed in response to the limited available length of the residual vasa deferentia and the anatomical and technical constraints encountered intraoperatively, rather than as a general alternative to conventional lateral abdominal wall fixation.

Potential complications specific to rectal wall fixation should also be considered. Inadvertent penetration of the rectal lumen during suture placement could result in local contamination and subsequent infection, whereas suture pull-through or tissue tearing could compromise fixation [15,18]. Fibrosis at the fixation site could theoretically restrict rectal distensibility or alter rectal motility, while repeated rectal distension and movement may place mechanical stress on the fixation site [15,19]. In the present case, partial-thickness rectal-wall bites incorporating the muscular layer were placed tangentially without penetration of the rectal lumen. However, these potential fixation-site complications could not be directly assessed postoperatively.

The local tissue condition and configuration of the pelvic diaphragm defects differed between the two sides, and identical reconstructive techniques were therefore not applied bilaterally. Internal obturator muscle transposition was performed on the left side, whereas on the right side, centrally located residual internal obturator muscle was preserved and the defects on either side were closed by apposition of the surrounding residual perineal tissues. Internal obturator muscle has also been incorporated into reconstruction during revision surgery for recurrent perineal hernia after previous internal obturator muscle transposition [20]. In the present case, the residual internal obturator muscle identified centrally was preserved and incorporated as part of the right-sided reconstruction. The sacrotuberous ligament was incorporated into selected sutures to reinforce the lateral defect [21].

Retrograde urethrocystography performed on postoperative day 8 demonstrated maintenance of the urinary bladder in its normal intra-abdominal position and restoration of the normal position and course of the bladder neck and urethra. These findings indicate that the short-term anatomical objectives of surgery were achieved. However, this result cannot be attributed solely to the vas deferens pexy, because bladder reduction following extensive adhesiolysis and bilateral pelvic diaphragm reconstruction likely contributed collectively to the short-term anatomical correction.

Despite this short-term anatomical correction, urinary function did not normalize immediately. Following removal of the urinary catheter on postoperative day 3, spontaneous urination was possible, but the urinary stream remained weak, and dysuria and urinary incontinence were observed. These abnormalities were still present 30 days postoperatively. By 150 days postoperatively, urinary function had gradually improved but had not completely normalized. Urinary abnormalities, including urine dribbling, stranguria, and urinary incontinence, have also been reported following surgical treatment of perineal hernia [6,14,22], indicating that urinary dysfunction may persist despite surgical treatment. In the present case, these abnormalities persisted despite documented short-term anatomical correction. Prolonged preoperative urinary retention may have resulted in secondary detrusor dysfunction or neural impairment that persisted after correction of the anatomical abnormalities, as functional recovery of the lower urinary tract may be delayed or incomplete following prolonged urinary retention or obstruction [23,24]. In addition, repeated pelvic and perineal surgeries and extensive dissection around the bladder neck and urethra may have contributed to postoperative urinary dysfunction [25]. However, functional urethral obstruction, detrusor dysfunction, or neurological dysfunction could not be objectively assessed because lower urinary tract functional testing was not performed. Fecal incontinence, which had been present preoperatively, persisted during the early postoperative period but had markedly improved by 150 days after surgery.

This case has several limitations. Detailed records of the previous surgeries were unavailable; therefore, the contribution of each previous procedure to recurrence and the development of extensive adhesions could not be assessed. In addition, the available length of the residual vasa deferentia and the tension on them after fixation were not objectively measured, and the potential reduction in additional dissection or operative time associated with rectal wall fixation was not quantified. The vas deferens–rectal wall fixation sites were not directly evaluated postoperatively; therefore, persistence of the fixation, the effects of rectal motility and filling on the fixation sites, and their long-term mechanical stability could not be determined. Objective assessment of urinary dysfunction during follow-up was limited because urinalysis, urine culture, measurement of residual urine volume, evaluation of bladder distension, and neurological examination were not performed. Defecatory function was also not objectively assessed. Furthermore, no additional diagnostic imaging was performed after postoperative day 8, preventing objective assessment of the long-term anatomical position of the urinary bladder, bladder neck, and urethra and the durability of rectal wall vas deferens pexy. Because recurrence of perineal hernia following internal obturator muscle transposition has been reported as late as approximately 1 year after surgery [22], the 150-day follow-up period was insufficient to assess long-term hernia recurrence.

## 4. Conclusions

In this case, a multimodal salvage procedure incorporating a combined perineal and abdominal approach, with perineal adhesiolysis performed before abdominal bladder reduction, and modified vas deferens pexy to the rectal wall was used in a dog with recurrent bilateral perineal hernia, extensive adhesions, and bladder retroflexion following repeated perineal and abdominal surgeries. Imaging performed 8 days postoperatively demonstrated short-term anatomical correction, with restoration of the normal anatomical positions of the urinary bladder, bladder neck, and urethra, although urinary function had not completely normalized by 150 days postoperatively. In the absence of further objective imaging, long-term mechanical stability and prevention of recurrence could not be established. This case illustrates the technical feasibility of tailoring the surgical sequence, organ stabilization technique, and pelvic diaphragm reconstruction according to intraoperative anatomy and tissue availability in selected salvage cases involving complex recurrent perineal hernia after multiple previous surgeries. Additional cases with long-term objective follow-up are required to evaluate the durability and clinical outcomes of rectal wall vas deferens pexy.

## Data Availability

The original contributions presented in this study are included in the article. Further inquiries can be directed to the corresponding author.
